# Determining the chemical activity of hydrophobic organic compounds in soil using polymer coated vials

**DOI:** 10.1186/1752-153X-2-8

**Published:** 2008-05-06

**Authors:** Fredrik Reichenberg, Foppe Smedes, Jan-Åke Jönsson, Philipp Mayer

**Affiliations:** 1 Department of Environmental Chemistry and Microbiology, National Environmental Research Institute, University of Aarhus, PO Box 358, 4000 Roskilde, Denmark; 2 Division of Analytical Chemistry, Lund University, PO Box 124, S-221 00 Lund, Sweden; 3 Ministry of Transport, Public Works and Water management, National Institute for Costal and Marine Management/RIKZ, PO Box 207, 9750 AE Haren, Netherlands

## Abstract

**Background:**

In soils contaminated by hydrophobic organic compounds, the concentrations are less indicative of potential exposure and distribution than are the associated chemical activities, fugacities and freely dissolved concentrations. The latter can be measured by diffusive sampling into thin layers of polymer, as in, for example, solid phase micro-extraction. Such measurements require equilibrium partitioning of analytes into the polymer while ensuring that the sample is not depleted. We introduce the validation of these requirements based on parallel sampling into polymer layers of different thicknesses.

**Results:**

Equilibrium sampling devices were made by coating glass vials internally with 3–12 μm thick layers of polydimethylsiloxane (PDMS). These were filled with slurries of a polluted soil and gently agitated for 5 days. The concentrations of 7 polycyclic aromatic hydrocarbons (PAHs) in the PDMS were measured. Validation confirmed fulfilment of the equilibrium sampling requirements and high measurement precision. Finally, chemical activities of the PAHs in the soil were determined from their concentrations and activity coefficients in the PDMS.

**Conclusion:**

PAHs' thermodynamic activities in a soil test material were determined via a method of uptake into PDMS. This can be used to assess chemical exposure and predict diffusion and partitioning processes.

## Background

Chemical activity and the closely related fugacity were suggested as measures of chemical behaviour and affinity by G. N. Lewis in his now century old formulation of chemical thermodynamics [1]. Since then they have been used to understand, predict and describe physico-chemical phenomena such as osmosis [2], mineral stability [3] and cholesterol biosynthesis [4,5]. That the chemical activity of a hydrophobic organic soil pollutant is important for its bioavailability has recently been proposed [6]. One reason for this is that partitioning occurs spontaneously down gradients in chemical activity. Equilibrium partitioning is defined by equal chemical activities, forming the basis for equilibrium partitioning theory [7] and related models (e.g. [8,9]). All of which is taken to contend that the mentioned thermodynamic quantities can help in the prediction of chemical bioaccumulation and toxicity [10]. Despite this promising perspective we found few published methods to determine the chemical activity [11,12] or fugacity [13-15] of hydrophobic organic pollutants in environmental samples.

In some instances chemical activities can be measured with equilibrium sampling techniques. One prominent approach, Solid Phase Micro-Extraction (SPME), was introduced in 1990 by Arthur and Pawliszyn [16]. The SPME format is very attractive as an Equilibrium Sampling Device (ESD) because the sampling phase is a small volume (μL) of polymer with a relatively large surface area [17]. The micro-extraction is caused by molecular diffusion across that surface, which is driven by the chemical activity of the analyte in the sample and results in a measurable concentration in the polymer. Diffusive sampling into thin layers of polymer is suitable for measurement of chemical activity and fugacity if certain conditions are fulfilled [17]. First, equilibrium (i.e. equal chemical activity) between the sample and polymer must be reached. Second, the uptake into the polymer must not deplete the sample of analyte, as this would reduce the chemical activity in the sample [18]. Third, polymer surface adsorption must be negligible because fugacity and activity is only proportional to the absorbed (dissolved) concentration within the polymer [19]. This proportionality links an observable, physically real concentration, to the abstract but meaningful thermodynamic activity function. The chemical activity (*a*) is thus given by:

(1) *a *= γ_P_·*C*_P _

where γ denotes the activity coefficient and *C *the concentration of the analyte in the polymer (subscript _P_). At equilibrium, the analyte concentration in the polymer reveals new and important information about the sample.

Clearly, when compared to the more traditional analytical extraction techniques, equilibrium sampling differs in both means and ends. Not surprisingly they also present other challenges, and method performance must be evaluated differently. For example, Soxhlet extractions used in quantitative analytical methods are designed for complete analyte recovery, and tend to be sample destructive yielding "dirty" extracts that require further work-up before analysis. For such exhaustive extractions, it is well established that analyte recovery during extraction and clean-up can be validated by internal standards. Equilibrium sampling, in contrast, dictates that the sample must remain intact during the procedure. For example, grinding [20], heating and soaking soil samples in organic solvents are to be avoided as far as they destroy the matrix and might alter the chemical activity to be measured. Likewise, it must be assured that the ESD polymer is not damaged by abrasion, or its surface fouled by soil particles during the agitated equilibration procedure. These special criteria for valid equilibrium sampling are known, but inadequately addressed in practice.

Equilibrium sampling has some unusual criteria, and reliability could be gained from an integrated validation of (1st) equilibrium, (2nd) negligible depletion of sample and (3rd) negligible impact of other experimental artefacts such as adsorption on the polymer surface. This is especially important when long equilibration times (days) make separate validation experiments costly or the polymer phase is given a large surface area to volume (A/V) ratio to speed up equilibration. However, we could not find any published equilibrium sampling methods able to fulfil these validation requirements.

Diffusive samplers with multiple A/V ratios can be used to confirm equilibrium without time series measurements [17,21]. Mechanistic arguments show that the A/V-approach allows not only validation of equilibrium, but extends to disclosing other potential artefacts such as sample depletion and polymer surface adsorption. The subsequent discussion explains the benefits of equilibrium sampling with devices of multiple coating thicknesses.

**Figure 1:**
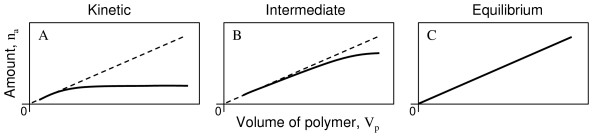
Schematic representation of the amount of analyte (n_a_) in thin layer ESDs as a function of their polymer volume (V_p_) at two time points before and at equilibrium. Dashed line represents equilibrium conditions.

Consider the case of parallel sampling with several ESDs having different polymer volumes (V_p_) but similar effective surface areas. In the kinetic uptake regime, the amount of analyte (n_a_) in the polymer results from a diffusive mass transfer proportional to the surface area, but independent of the polymer volume (Figure 1A). In the intermediate uptake regime, back-diffusion out of the polymer becomes increasingly significant, and n_a _depends then on both the surface area and V_p _(Figure 1B). At equilibrium, n_a _is proportional to V_p _(Figure 1C), which allows the confirmation of equilibrium without studying up-take kinetics while also providing the measurements needed for the calculation of mean concentration and standard deviation. This economises time and analytical resources by allowing the same data both to confirm and measure equilibrium conditions.

**Figure 2:**
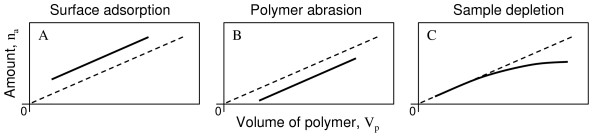
Schematic representation of the amount of analyte (n_a_) in/on thin layer ESDs as a function of their polymer volume (V_p_) in three cases of equilibrium sampling artefact. Dashed line represents artefact-free conditions.

Equilibrium sampling with multiple polymer coating thicknesses can also reveal various sampling artefacts such as surface adsorption, polymer abrasion and sample depletion. Proportionality between n_a _and V_P _confirms valid sampling (dashed line, Figure 2). Surface adsorption would contribute an extra amount to n_a _independently of the polymer volume and to a positive bias on the intercept (Figure 2A). Abrasive loss of polymer, that in our experience predominantly befall thin layers, would lead to a reduced n_a _compared to the artefact free situation (Figure 2B). Sample depletion, meaning a significant impact from analyte mass-transfer during equilibration, would lead to a reduction of the analyte concentration with increasing V_P _and to curving in Figure 2C. Other and more complicated situations can easily be imagined. However, if the measured amounts, when plotted as in Figure 2, can be connected by a straight line passing through the origin, this is a strong indication of a successful and valid equilibrium sampling.

Many different ESD formats have been applied, including PDMS coated glass fibres [22], low-density polyethylene strips [23,24], polyoxymethylene strips [25], poly(ethylene-vinyl acetate) films [14] and liquid-filled, hollow-fibre membranes [26], each with their specific operational window. Other alternatives include the Twister^® ^(sorptive stir-bar) and the Immobilised Liquid Extraction™ Caps. Very recently Minhas et al. published work on vials internally coated with poly(ethylene-vinyl acetate) [27]. This integration of glass container with the sampling phase allows soil and sediment samples to be transported and stored, for example, while equilibrating with the polymer coating. All the while minimising sample handling, increasing work efficiency and reducing worker exposure.

The limitations of existing ESDs and theoretical arguments suggest that equilibrium sampling with multiple coating thicknesses integrated within a sample container can provide validated and simplified measurements of chemical activity, fugacity and freely dissolved concentration. The aim of this paper is therefore to present ESDs consisting of μm-thick PDMS coatings of multiple thicknesses and their application to the measurement of chemical activity of PAHs in soil.

### Working principle

The ESDs consist of PDMS layers deposited on the internal wall of sample glass vials. The ESDs are equivalent in all respects apart from the thickness of the PDMS. This in vial-sample preparation minimises sample handling, and parallel application of the different thicknesses allows validation of the equilibrium sampling to measure chemical activity. The procedure consists of three parts:

1. Vials with 3–12 μm thin layers of PDMS are equilibrated with samples of the investigated soil.

2. Solvent extracts of the PDMS are analysed for the content of PAHs to determine *C*_PDMS_.

3. The chemical activity is found as the product of *C*_PDMS _and an activity coefficient (Equation 1).

## Results and discussion

### Determining equilibrium partitioning concentrations in PDMS coatings

**Figure 3:**
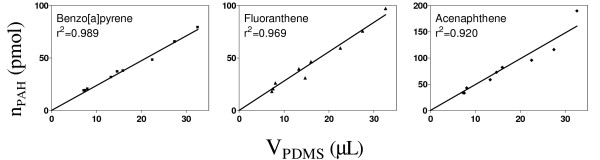
Amounts of analyte PAH (n_PAH_) plotted as a function of volume of PDMS (V_PDMS_) in nine coated vials after equilibration with the test soil (in slurry) for five days. Three representative compounds were selected for having the highest, median and lowest r^2^-values.

After sampling the soil in PDMS coated vials, the quantities of PAHs (n_PAH_) in the PDMS were extracted to methanol, measured by HPLC, and plotted against the PDMS volumes (Figure 3). Linear regressions (least square fit, forced [0;0]) with r^2 ^exceeding 0.90 were obtained for 7 PAHs. The graphs clearly demonstrate that n_PAH _is proportional to V_PDMS_, which is consistent with artefact-free equilibrium sampling.

Equilibrium partitioning concentrations in the PDMS (*C*_PDMS_, Table 1) were calculated as the arithmetic mean of the 9 coated vials. The relative standard error of the arithmetic mean ranged from 2 to 4 %, a very high precision. Additionally, *C*_PDMS _was also determined as the slope of the linear regression. These slope estimates and the arithmetic means hardly differed (2–4 %), and both calculation principles are equally suitable. A high accuracy in the *C*_PDMS _measurements is expected, since the potential for systematic errors associated with the input parameters (n_PAH_, V_MeOH_, V_PDMS_) are limited.

**Table 1:** Measured equilibrium partitioning concentration in PDMS (*C*_PDMS_), chemical activity (*a*) and concentration (*C*_soil_) of seven PAHs in the test soil

**Compound**	***C*_PDMS _(± SD)^*a*^**		***a*_soil_**	***C*_soil_**
	μ**M**		**10^-6^**	**mg/kg**^*b*^

Ace	4.42	(± 0.17)	13	1
Ant	1.51	(± 0.05)	8.6	5
Flu	2.55	(± 0.10)	18	17
BaA	1.50	(± 0.04)	23	10
BkF	1.07	(± 0.03)	35	8
BaP	2.23	(± 0.04)	62	9
Ind	1.81	(± 0.07)	151	7
				
^*a *^*n *= 9-8				
^*b *^dry matter				

The method limit of quantification (LoQ) was defined as 10 times the standard deviation of the signal noise level. In terms of *C*_PDMS_, LoQ ranged from 0.1 to 2.5 μM (Table 2), and varied with the PAHs' specific fluorescence quantum yield at the detector settings of the HPLC. There are several ways to lower the detection limits, including evaporative concentration of the methanol extract and the use of more sensitive detectors. If analytical procedures require, the vials may be extracted with other solvents than methanol (e.g. pentane). This may require a more rigorous pre-extraction of siloxane oligomers from the coatings to keep these from co-extracting with the analytes [28].

**Table 2:** Method Limits of Quantification (LoQ)

	**LoQ**			
**Compound**	***C*_MeOH_**	***C*_PDMS_**	***a*_soil_**	**Depletion of sample**

	**nM**	μ**M**	**10^-6^**	**%**

Nap	19.5	2.5	2.6	
Ace	4.1	0.5	1.5	0.26
Flo	11.6	1.5	3.2	
Phe	6.0	0.8	3.1	
Ant	1.8	0.2	1.3	0.01
Flu	12.4	1.6	11.2	0.01
Pyr	6.5	0.8	3.1	
BaA	2.7	0.3	5.3	0.01
Chr	6.3	0.8	21.6	
BkF	0.7	0.1	2.9	0.01
BaP	0.8	0.1	2.8	0.02
Ind	12.9	1.6	137	0.02

Values of *C*_PDMS _can be applied in many different ways without any further conversion. Gradients in *C*_PDMS _follow the potential for diffusion and measured values should fall in the direction of passive transport gradients within or between environmental compartments. For example, as predictor of PCB bioaccumulation into worms, *C*_PDMS _is directly comparable between both dissimilar sediments [29] and soils [30]. Likewise, *C*_PDMS _can be used to monitor environmental processes in time, such as the effect of aging or the (bio)degradation of PAHs in soil. A decrease in *C*_PDMS _is sufficient to show a decrease in chemical activity.

### Determining freely dissolved concentration and chemical activity

*C*_PDMS _is proportional to the freely dissolved concentration (*C*_free_), chemical activity and also fugacity [6]. *C*_free _can be determined with the analyte specific PDMS-water partition ratio *K*_PDMS/aq_:

(2) $$C_{free}=\frac{C_{PDMS}}{K_{PDMS/aq}}$$

This calibration towards *C*_free _has already been covered in several publications (e.g. [17,22,31,32]), and the present paper focuses on the determination of chemical activities from *C*_PDMS _(Equation 1).

### Activity coefficients in PDMS

The activity coefficients in Table 3 were determined at solubility (Equation 3). At this limit, the chemical activity of the solute PAH in the saturated solvent is usually equal to the activity of the PAH in its pure solid crystal state, *a*_xstal_. In turn, *a*_xstal _is exponentially related to the free energy difference between the compound's liquid and solid states at the given temperature. This difference can be determined from the melting point temperature, heat of fusion and heat capacity as detailed elsewhere [33,34]. However, *a*_xstal _were instead estimated through Equation 4, for reasons of consistency. For similar reasons, the activity coefficients were based on solubility in methanol and the partition ratio to the PDMS. First, S_(s)MeOH _and *K*_PDMS/MeOH _could be determined with sufficient precision (Table 3 and Table A in Additional File 1). Second, S_(s)MeOH _for several PAHs are published and can be used to validate experimental accuracy (Figure A in Additional File 1). Third, the use of partitioning solutions and solubilities in the calibration of ESDs is consistent with current practice [5,11,15,17,22,35] and carries certain advantages.

**Table 3:** Melting points (T_m_), crystal activities (*a*_xstal_), solubilities (S_(s)MeOH_) and activity coefficients (γ_MeOH_) in methanol. Methanol-PDMS^e ^partition ratios (*K*_PDMS/MeOH_) and activity coefficients (γ_PDMS_) in the PDMS for 12 PAHs

**Compound**	**T_m_**	***a*_xstal_^*a, b*^**	**S_(s)MeOH _(± SD)^*b, c*^**		γ**_MeOH_^*d*^**	***K*_PDMS/MeOH_^*e*^**	γ**_PDMS_^*e, f*^**
	**K**		**mM**		**M^-1^**	**LL^-1^**	**M^-1^**
Nap	354	0.288	500	(± 23.5)	0.6	0.56	1.0
Ace	367	0.210	103.20	(± 0.82)	2.0	0.68	3.0
Flo	385	0.131	114.63	(± 2.41)	1.1	0.53	2.2
Phe	372	0.188	112.43	(± 6.51)	1.7	0.41	4.1
Ant	489	0.013	6.12	(± 0.13)	2.2	0.38	5.7
Flu	383	0.146	53.42	(± 5.05)	2.7	0.38	7.1
Pyr	425	0.058	31.64	(± 0.39)	1.8	0.49	3.8
BaA	433	0.047	10.37	(± 0.47)	4.6	0.30	15.0
Chr	529	0.005	0.70	(± 0.02)	7.8	0.29	27.1
BkF	489	0.013	1.49	(± 0.11)	8.9	0.27	32.6
BaP	454	0.029	3.49	(± 0.06)	8.4	0.30	27.9
Ind	435	0.045	1.64	(± 0.03)	27.6	0.33	83.5
							
^*a *^Equation 4							
^*b *^T = 298 K (25°C)							
^*c *^*n *= 3							
^*d *^γ_MeOH _= *a*_xstal_S_(s)MeOH_^-1^							
^*e *^Silastic^® ^PDMS							
^*f *^Equation 3							

The practice of ESD calibration via methanol partitioning standards may prove valuable in the future. Already now, methods to conduct multi-compartment studies including equilibrium sampling measurements are available [14,22,23,36]. However, these methods use different polymers and this may confound a comparison of results. For example, the inter-calibration of two commercial PDMS formulations, Silastic^® ^and SSP-M823, revealed an almost 40% difference in their partitioning properties (Table A in Additional File 1). This kind of information is important for comparing measurements from different ESDs.

### Equilibrium sampling devices with multiple A/V ratios

The in vial-ESD format and A/V-approach has unique and important features. Most important is the integrated QA/QC of the measurements. This extends the applicability towards very difficult or very dirty samples that might cause problems during equilibration or analysis due to their composition. Examples of troublesome sample components include soot particles with a high PAH content [25] and (micro)droplets of non-aqueous phase liquids (NAPLs) that may foul the PDMS surface. But, when using the in vial-ESD format, equilibrium sampling flaws can be inspected by plotting n_a _against V_PDMS_. This integrated validation makes the method reliable at its applicability limit, whereas other equilibrium sampling methods require a greater margin of safety. Second, the integration of thin polymer layers within sample vials [27] reduces the risk of measurement error. Handling procedures are minimised as sample material can be secured, transported, stored and equilibrated, under seal and in a single container. The subsequent liquid extract of the PDMS makes the method compatible with many standard analytical procedures. For example, addition of internal standards, clean-up and/or pre-concentration steps can be included in future equilibrium sampling method developments. The in vial-ESDs are both practical and versatile. Third, the design of the devices is also adaptable. The ESD dimensions can be tuned towards faster equilibration by reducing the polymer coating thickness [27]. Alternatively, detection limits can be lowered by increasing the V_PDMS _to sample a greater amount of analyte for detection. All of these advantages make ESDs with multiple A/V ratios attractive.

## Experimental

### In vial-ESD preparation

3–12 μm layers of PDMS were prepared in 20 mL glass vials by dispersion coating in the following manner. The appropriate mass of medical adhesive, Silastic^® ^PDMS Silicone Type A (Dow Corning, Seneffe, BE; density 1.15 kg/L [28]) paste was dispersed in 10 times its volume in n-pentane by wrist shaking and sonication. The dispersion was filtered (glass fiber, 0.7 μm nominal, Pall Corp., MI) and diluted in a geometric series. All polymer dispersions were used at the day of preparation and should be prepared using disposable glassware and materials to avoid inadvertent formation of insoluble polymer layers.

After careful tare on an analytical balance (Sartorius BP 210S, d = 0.1 mg), the vials to be coated were placed horizontally on a roller-mixer (Stuart sci., Stone, UK). While rotated (33 rpm) 3.0 mL of the appropriate dispersion was added to each vial. The solvent was evaporated under a gentle stream of nitrogen and the vials were left to polymerise for 72 h in a fume hood. Siloxane oligomers were removed from the cross-linked PDMS by three extractions with ethyl acetate (3 mL, 10 min vigorous shaking). The vials were again balanced to determine the mass of PDMS now covering their inner sidewall surface.

### Equilibrium sampling procedure

PDMS-coated vials with different coating thicknesses were filled with 18–20 g of the test soil (*n *= 9). 10 mL aqueous solution of sodium azide (0.5 g/L) was added to each vial to inhibit microbial activity and to create a soil suspension for the sampling. Vials were capped with metal lined screw caps and placed horizontally in a customised holder rotated (6 rpm) on a roller-mixer for 122 h.

### PDMS coating extraction

Soil content was removed, the vials were rinsed with a little distilled water on a whirly mixer before being wiped internally with lint-free tissue. To each vial was then added 1.00 mL methanol. The vials were again capped and rotated for 12 h. The methanol extracts were collected and stored in freezer (-20°C) until analysed for PAHs.

### Extract analysis

Methanol extracts of PDMS were analysed for PAHs by HPLC-fluorescence detection (Agilent 1100 system with G1321A FLD (Ex. 260 nm; Em. 350, 420, 440 and 500 nm). Separation column: CP-Ecospher 4 PAH (Varian Inc., Palo Alto, CA) operated at 0.5 ml/min (28°C, 10 μL injection); Mobile phase: methanol, SUPER-Q treated water (Millipore, MA); Gradient (in %methanol, by weight): t = 0 min 80%; t = 5–30 min linear gradient 80–100%; t = 30–45 min 100%. Quantification was accomplished by a five-point external standard curve. Extract analysis was carried out within 2 weeks after sampling.

### Solubilities in methanol

Solubilities were measured by placing excess PAH crystals together with methanol in PTFE-sealed glass vials and equilibrating these in a thermostated water bath at 25.0 ± 0.1°C for at least 3 days. Attainment of the solubility limit was verified by repetitive measurements on consecutive days. Aliquots of the saturated methanol solutions were passed through 0.2 μm PTFE-filters into volumetric flasks and quantitatively diluted with methanol to reach suitable concentrations before analysis by the HPLC-FLD method described above.

### PDMS-methanol partition ratios

Partition ratios (*K*_PDMS/MeOH_) between Silastic^® ^PDMS polymer and methanol was measured in a separate experiment. First, PDMS sheets were prepared by filling a polyethylene mold (0.25 mm high) with Silastic^® ^paste. The top surface was pressed even, and covered with wet tissue and a glass plate. After one week, the cross-linked PDMS was cut into sheets weighing approximately 1 g, Soxhlet extracted (ethyl acetate, 100 h) and then washed with methanol. A clean sheet and an excess of methanol solution containing all the analyte PAHs (approx. 25 μg/L) were placed in a sealed amber glass container. After equilibration by shaking for > 1 week at 20°C, the methanol and PDMS were separately taken for extraction and analysis of the PAH contents by GC-MS as described in [37]. The respective, volume based, concentrations in the Silastic^® ^PDMS sheet and the methanol solution were divided to calculate the partition ratios.

## Conclusion

This study concludes that vials containing thin layers of PDMS can be used as equilibrium sampling devices. The methodology of parallel sampling in devices with different thickness PDMS layers has unique and important features. Most important is an integrated QA/QC of the procedure. When conducted on a soil test material, results were confirmed as precise and valid measurements of equilibrium partitioning concentrations, *C*_PDMS_. These were then, by way of a separate calibration experiment, expressed as chemical activities of individual PAHs in the soil.

### Relevance for chemical risk assessment

A risk assessment of the pollution in the test material soil is warranted by the present soil quality criterion for PAHs in Denmark. The legislation dictates that the sum of fluoranthene, benzo [a]pyrene, benzo [k]fluoranthene, and indeno [1,2,3-cd]pyrene may not exceed 4 mg/kg dry soil [38].

Several concerns regarding the risk posed by the PAHs in the polluted soil can be assessed with the chemical activities reported in Table 1. First, *a*_soil _quantifies the chemical pollutant's potential for partitioning and distribution to other materials in contact with the soil. This makes the activity relevant because it is first after release and transfer to a susceptible target that a PAH may cause detriment of health or effects on the environment. Second, *a*_soil _provides a measure for comparison of chemical contamination levels. The activity reflects a chemical's affinity (e.g. partial molar free energy of sorption) to the soil material. Soils are dynamic, heterogeneous and unpredictable geosorbents. Therefore, *C*_soil _is harder to interpret and compare than are concentrations in a well-defined, stable and homogenous phase such as PDMS. In consequence, equilibrium sampling measurements are useful because the results can easily be compared to the levels in other measured soils, in air, water or biological tissue, as well as to effect levels determined in toxicity studies, whenever such are available.

**Figure 4:**
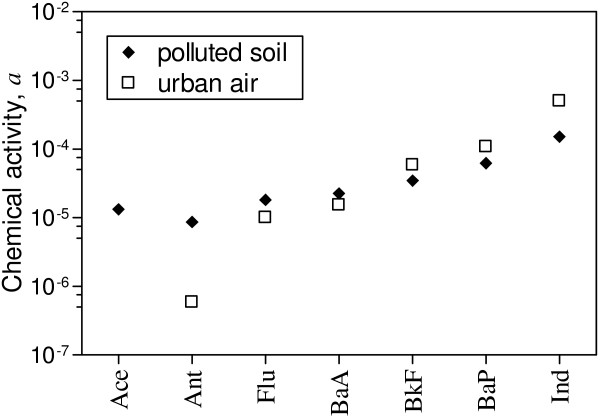
Chemical activities in the test soil (◆) compared to activities in the gas phase of Heraklion (GR) inner city air (□). The latter are based (see Additional File 1) on gas phase concentrations, measured by high-volume sampling and reported in [39].

For example, comparison of different exposure routes may be part of health risk assessments. Cases may include, for instance, risk of systemic up-take after ingestion of polluted soil and inhalation of urban air. To illustrate, Figure 4 depicts chemical activities of several priority pollutant PAHs, both in the tested soil and in the gas phase of an urban atmosphere [39](Additional File 1). Comparatively, PAHs in the air seem to have greater chemical potential for diffusion through biological membranes, such as gastrointestinal or alveolar epithelium.

## Methods

### Standards

PAHs of ≥98% purity were obtained from Sigma-Aldrich (Copenhagen, DK) or Cambridge Isotope Lab. (Andover, MA).

### Solvents and chemicals

P.a. grade n-pentane, ethyl acetate, sodium azide and HPLC-grade methanol were obtained from Merck (Darmstadt, DE).

### Soil material

The PAH contaminated soil were excavated from a former manufactured gas plant site (Gaswerk Tiefstark, Klosterwall 2, Freie Hansestadt Hamburg, DE) and then compost treated by A/S Bioteknisk Jordrens (Esbjerg, DK) prior to this study. It contained 14 % water, 5–7 % organic carbon, 11–16 % silt, 3–4 % clay and weathered coal-tar, petroleum residues, soot and ash.

The bioremediation of the soil had removed a fraction of the PAHs; whereas those associated with the black carbon [40] likely remained. The residual total concentration of the 16 EPA PAHs was 120 mg/kg_soil_. This test material was selected on account of the PAH-containing soot that is claimed to complicate equilibrium sampling [25]. Prior to sampling, the soil was sieved to 2 mm (steel mesh) and manually homogenized.

### QA/QC and statistical treatment

The qualification criteria for PAHs extracted from the ESDs included retention times within 5% of a known sample and correct fluorescence emission wavelength(s). Signal integration was performed with HP Chemstation software (A.06.03, Agilent Tech.) and corrected by hand as necessary. About 7% standards and solvent blanks were included in the analytical sample sequences. ESD blanks were considered unnecessary since positive blank values would be eliminated during the equilibration. The measured results were tested by least squares linear regression of n_PAH _on V_PDMS_, forced through the origin. An r^2 ^> 0.90 goodness of fit, was set as a necessary but not sufficient equilibrium sampling criterion. One outlier in the data set for anthracene was defined as the measured value of n_ANT _differed by a factor > 2 from the predicted (*n *= 9). Equilibrium concentrations in PDMS (*C*_PDMS_, Table 1) were calculated as the arithmetic mean of the measurements (*n *= 9-8).

### Activity coefficients in PDMS

For each PAH in Table 3, the activity coefficient in the Silastic^® ^PDMS (γ_PDMS_) that coated the vials was determined from partitioning and solubility data:

(3) $$\gamma_{PDMS}=\frac{a_{xstal}}{S_{(s)MeOH}K_{PDMS/MeOH}},$$

where *a*_xstal _is the activity of the PAH in its crystal state, S_(s)MeOH _the solubility in methanol and *K*_PDMS/MeOH _is the Silastic^® ^PDMS-methanol partition ratio.

### Crystal activities

The PAHs' activities in their pure solid crystals, also known as fugacity ratios [41], at 25°C (T = 298 K) were estimated from melting point temperatures (T_m_, K), assuming their entropy of melting to be 56 Jmole^-1^K^-1 ^(i.e. Walden's rule) as suggested by Yalkowsky et al. [42]:

(4) $$a_{xstal}=\exp(6.8\left[1-\frac{T_{m}}{T}\right])$$

## List of abbreviations

See table 4.

**Table 4:** List of abbreviations

		**InChI**
Nap	Naphthalene	InChI=1/C10H4Cl4/c11-7-5-3-1-2-4-6(5)8(12)10(14)9(7)13/h1-4H
Ace	Acenaphthene	InChI=1/C12H10/c1-3-9-4-2-6-11-8-7-10(5-1)12(9)11/h1-6H,7-8H2
Flo	Fluorene	InChI=1/C13H10/c1-3-7-12-10(5-1)9-11-6-2-4-8-13(11)12/h1-8H,9H2
Phe	Phenanthrene	InChI=1/C14H10/c1-3-7-13-11(5-1)9-10-12-6-2-4-8-14(12)13/h1-10H
Ant	Anthracene	InChI=1/C14H10/c1-2-6-12-10-14-8-4-3-7-13(14)9-11(12)5-1/h1-10H
Flu	Fluoranthene	InChI=1/C16H10/c1-2-8-13-12(7-1)14-9-3-5-11-6-4-10-15(13)16(11)14/h1-10H
Pyr	Pyrene	InChI=1/C16H10/c1-3-11-7-9-13-5-2-6-14-10-8-12(4-1)15(11)16(13)14/h1-10H
BaA	Benzo [a]anthracene	InChI=1/C18H12/c1-2-7-15-12-18-16(11-14(15)6-1)10-9-13-5-3-4-8-17(13)18/h1-12H
Chr	Chrysene	InChI=1/C18H12/c1-3-7-15-13(5-1)9-11-18-16-8-4-2-6-14(16)10-12-17(15)18/h1-12H
BkF	Benzo [k]fluoranthene	InChI=1/C20H12/c1-2-6-15-12-19-17-10-4-8-13-7-3-9-16(20(13)17)18(19)11-14(15)5-1/h1-12H
BaP	Benzo [a]pyrene	InChI=1/C20H12/c1-2-7-17-15(4-1)12-16-9-8-13-5-3-6-14-10-11-18(17)20(16)19(13)14/h1-12H
Ind	Indeno [1,2,3-cd]pyrene	InChI=1/C22H12/c1-2-7-17-16(6-1)18-11-10-14-9-8-13-4-3-5-15-12-19(17)22(18)21(14)20(13)15/h1-12H
		
PAH		Polycyclic Aromatic Hydrocarbon
PDMS		PolyDiMethylSiloxane
MeOH		Methanol
aq		Water
free		Water
p		Polymer
		
		**Unit**
SPME	Solid Phase Micro-Extraction	
ESD	Eqilibrium Sampling Device	
A/V	(surface)Area to Volume ratio	m^-1^
LoQ	Limit of Quantification	
QA/QC	Quality Assurance/Quality Control	
PTFE	PolyTetraFluoroEthylene	
		
*a*	Chemical (thermodynamic) activity	
γ_i_	Activity coefficient in phase i	M^-1^
*K*_i/j_	Partition ratio between phases i and j	L/L
*C*_i_	Concentration in (phase) i	M
T	Thermodynamic temperature	K
T_m_	Melting point temperature	K
V_i_	Volume of phase i	L
n_a_	Amount of analyte	mol
S_(s)i_	Solubility (solid) in phase i	M
*n*	number of replicate determinations	
r^2^	Goodness of fit	

## Authors' contributions

FR carried out ESD preparation, sampling and analysis; structured and wrote the manuscript. FS developed ESD preparation method and measured *K*_PDMS/MeOH_. JJ participated in the design of the study. PM conceived the idea, shared in its design and evaluation; structured and wrote the manuscript. All authors read and approved the final manuscript.

## Supplementary Material

Additional File 1 Graph comparing published values of S_(s)MeOH _for a number of PAHs; an additional experimental determination of *K*_PDMS/MeOH_; an estimation of PAH's activities in urban air. Click here for file
