# Supplementary material for: Determining the chemical activity of hydrophobic organic compounds in soil using polymer coated vials
Source: Chem Cent J. 2008 May 6;2:8. doi: 10.1186/1752-153X-2-8 (PMC2409337; doi:10.1186/1752-153X-2-8)
Supplement: Additional File 1 — Graph comparing published values of S(s)MeOH for a number of PAHs; an additional experimental determination of KPDMS/MeOH; an estimation of PAH's activities in urban air. [file 1752-153X-2-8-S1.doc]

**Additional file 1**

**Figure A.** Solid solubilities of PAHs in methanol (S_s,MeoH_, 25°C) from Table 1 (■) and values reported in literature (**×**) [1-3] as a function of compound melting temperature (T_m_).

**An alternative determination of *K*_PDMS/MeOH_**.

The partition ratios between methanol and two different commercial PDMS formulations were determined simultaneously. First, eight pieces (á 2.0±0.1g) of PDMS membrane (SSP-M823, density 1.17g/mL, Specialty Silicone Products, inc.) were placed in excess methanol solution containing all the analyte PAHs. The solution was gently stirred for 12 hours to equilibrate [4]. Samples of the methanol standard were taken; the equilibrated membranes were wiped dry on lint-free tissue. Four of them were immediately extracted with methanol (200 mL) while the remaining were placed on the Silastic® PDMS (<50 mg) layer in four coated vials (ESDs). These were sealed and kept in darkness at room temperature for 8 days before the membranes were removed and the vials were extracted in methanol as above. All the PDMS extracts were analyzed for PAHs together with samples of the methanol standard solution. The PAH concentrations in the two phases were divided to calculate values of *K*_PDMS/MeOH_. Results are presented in Table A.

**Table A.** Concentrations in methanol partitioning standard (*C*_MeOH_, *n*=6) and partitioning equilibrium concentrations in two commercial PDMS materials; SSP membranes (*C*_SSP_, *n*=4) and Silastic® coatings (*C*_Sil_, *n*=4) for 13 PAHs. From this partition ratios (*K*) were calculated.

| **Compound** | ***C*_MeOH_ (±SD)** | |  | ***C*_SSP_ (±SD)** | |  | ***C*_Sil_ (±SD)** | |  | ***K*_SSP/MeOH_** | ***K*_Sil/MeOH_** |
| --- | --- | --- | --- | --- | --- | --- | --- | --- | --- | --- | --- |
|  | **μg/L** | | | | | | | |  | **L/L** | |
| Napthalene | 5559 | (±222) |  | 2171 | (±48) |  | 2902 | (±81) |  | 0.39 | 0.52 |
| Acenapthene | 5994 | (±270) |  | 3306 | (±82) |  | 4513 | (±59) |  | 0.55 | 0.75 |
| Fluorene | 6111 | (±269) |  | 2581 | (±70) |  | 3442 | (±50) |  | 0.42 | 0.56 |
| Phenanthrene | 6526 | (±264) |  | 1999 | (±63) |  | 2690 | (±48) |  | 0.31 | 0.41 |
| Anthracene | 6823 | (±248) |  | 2217 | (±58) |  | 2922 | (±64) |  | 0.32 | 0.43 |
| Fluoranthene | 5802 | (±193) |  | 1701 | (±57) |  | 2260 | (±80) |  | 0.29 | 0.39 |
| Pyrene | 5741 | (±106) |  | 1845 | (±76) |  | 2420 | (±66) |  | 0.32 | 0.42 |
| Benz(a)anthracene | 5714 | (±112) |  | 1443 | (±53) |  | 1836 | (±19) |  | 0.25 | 0.32 |
| Chrysene | 6651 | (±118) |  | 1640 | (±68) |  | 2087 | (±27) |  | 0.25 | 0.31 |
| Benzo(b)fluoranthene | 6339 | (±123) |  | 1452 | (±52) |  | 1852 | (±45) |  | 0.23 | 0.29 |
| Benzo(k)fluoranthene | 5941 | (±113) |  | 1453 | (±51) |  | 1781 | (±40) |  | 0.24 | 0.30 |
| Benzo(a)pyrene | 6212 | (±98) |  | 1600 | (±57) |  | 1962 | (±44) |  | 0.26 | 0.32 |
| Indeno(1,2,3-cd)pyrene | 6487 | (±118) |  | 1583 | (±88) |  | 2039 | (±28) |  | 0.24 | 0.31 |

**PAH activities in urban air.**

Tsapakis et al. [5] measured gas-phase concentrations (*C*_gas_, g/m^3^) of PAHs in urban air (Heraklion, GR). By some approximation these were converted to partial pressures or chemical activities through:

$a_{\mathrm{air}}=\frac{C_{gas}\mathrm{RT}}{\mathrm{MP}_{\mathrm{L}}^{\mathrm{o}}}$ (A),

where R (8.314 Jmole^-1^ K^-1^) is the gas constant, M (g/mol) the molar mass of the PAH and P^o^_L_ (Pa, 298 K, values taken from [6]) is its (hypothetical) liquid vapor pressure. As a consequence, *a*_air_ is given with reference to the pure PAH liquid state. The air temperature (T) was set constant to 298 K, thus neglecting the 9-26 °C variability.

As *C*_gas_ input data for Figure 4 we used mean values reported from >1 year of weekly measurements [5].

# References

1. Pinal R, Rao PSC, Lee LS, Cline PV, Yalkowsky SH: Cosolvency of partially miscible organic-solvents on the solubility of hydrophobic organic-chemicals. *Environ Sci Technol* 1990, 24:639-647.

2. Hernandez CE, Acree WE: Solubility of fluoranthene in organic nonelectrolyte solvents. Comparison of observed versus predicted values based upon mobile order theory. *Can J Chem* 1998, 76:1312-1316.

3. Fan CH, Jafvert CT: Margules equations applied to PAH solubilities in alcohol-water mixtures. *Environ Sci Technol* 1997, 31:3516-3522.

4. Booij K, Smedes F, van Weerlee EM: Spiking of performance reference compounds in low density polyethylene and silicone passive water samplers. *Chemosphere* 2002, 46:1157-1161.

5. Tsapakis M, Stephanou EG: Occurrence of gaseous and particulate polycyclic aromatic hydrocarbons in the urban atmosphere: Study of sources and ambient temperature effect on the gas/particle concentration and distribution. *Environ Pollut* 2005, 133:147-156.

6. Odabasi M, Cetin E, Sofuoglu A: Determination of octanol-air partition coefficients and supercooled liquid vapor pressures of pahs as a function of temperature: Application to gas-particle partitioning in an urban atmosphere. *Atmos Environ* 2006, 40:6615-6625.
